# Assessing the Usefulness of Interleukin-8 as a Biomarker of Inflammation and Metabolic Dysregulation in Dairy Cows

**DOI:** 10.3390/ijms252011129

**Published:** 2024-10-16

**Authors:** Kamila Puppel, Jan Slósarz, Paweł Solarczyk, Grzegorz Grodkowski, Piotr Kostusiak, Aleksandra Kalińska, Marek Balcerak, Małgorzata Kunowska-Slósarz, Marcin Gołębiewski

**Affiliations:** Institute of Animal Science, Warsaw University of Life Sciences, Ciszewskiego 8, 02-786 Warsaw, Poland; jan_slosarz@sggw.edu.pl (J.S.); pawel_solarczyk@sggw.edu.pl (P.S.); grzegorz_grodkowski@sggw.edu.pl (G.G.); piotr_kostusiak@sggw.edu.pl (P.K.); aleksandra_kalinska@sggw.edu.pl (A.K.); marek_balcerak@sggw.edu.pl (M.B.); malgorzata_kunowska_slosarz@sggw.edu.pl (M.K.-S.); marcin_golebiewski@sggw.edu.pl (M.G.)

**Keywords:** interleukin-8, SCC, GGTP, AspAT, dairy cows, inflammation, metabolic health, mastitis, ketosis

## Abstract

The study aimed to evaluate interleukin-8 (IL-8) as a biomarker for udder inflammation in dairy cows and to explore its associations with various metabolic parameters indicative of systemic inflammation and metabolic dysregulation. Dairy cows (multiparous) were categorized into five somatic cell count (SCC) classes: Class I (<100,000 cells/mL; n = 45), Class II (100,000–200,000 cells/mL; n = 62), Class III (201,000–400,000 cells/mL; n = 52), Class IV (401,000–1,000,000 cells/mL; n = 73), and Class V (>1,000,000 cells/mL; n = 56). The study quantified IL-8 levels and analyzed their correlations with NEFAs (non-esterified fatty acids), BHBA (beta-hydroxybutyrate), GGTP (gamma-glutamyltransferase), and AspAT (aspartate aminotransferase). IL-8 concentrations demonstrated a significant and progressive increase across the SCC classes, establishing a strong positive correlation with SCC (*p* < 0.01). Additionally, IL-8 levels exhibited positive correlations with GGTP (*p* < 0.01) and AspAT (*p* < 0.01), indicating that elevated IL-8 is associated with increased hepatic enzyme activities and potential liver dysfunction. Furthermore, IL-8 showed significant positive correlations with NEFAs (*p* < 0.01) and BHBA (*p* < 0.05), linking higher IL-8 levels to metabolic disturbances such as ketosis and negative energy balance. Variations in metabolic parameters, including NEFAs, BHBA, GGTP, and AspAT, across the SCC classes underscored the association between elevated SCC levels and metabolic dysregulation in dairy cows. These findings highlight the interrelated nature of the inflammatory responses and metabolic disturbances in dairy cattle, emphasizing that an elevated SCC not only signifies udder inflammation but also correlates with systemic metabolic alterations indicative of ketosis and liver damage.

## 1. Introduction

Interleukin-8 plays a pivotal role in the inflammatory response as a major neutrophil chemoattractant. IL-8, a chemokine produced by various cells including endothelial cells and neutrophils themselves, specifically attracts neutrophils to the sites of infection or injury, and it does not influence monocytes or lymphocytes [1,2]. The binding of IL-8 to its receptors on neutrophils activates G protein-coupled signaling pathways that lead to neutrophil migration, adhesion to the endothelium, and subsequent activation [3]. Neutrophils respond to IL-8 through a series of cellular processes including adherence to the endothelial cell layer and transendothelial migration toward the inflammatory site [4]. IL-8 is synthesized in response to inflammatory stimuli such as IL-1 or lipopolysaccharides (LPS) and is stored in Weibel-Palade bodies within endothelial cells, where it is released upon stimulation [5]. Once in the bloodstream, IL-8 undergoes transcytosis and is presented at the tips of microvilli on the luminal surface of endothelial cells, where it facilitates the recruitment of neutrophils [6]. Additionally, IL-8 serves not only as a chemoattractant but also as a priming agent for neutrophils. It enhances neutrophil responses such as the production of reactive oxygen species (ROS) and degranulation, which are crucial for the elimination of pathogens and the resolution of inflammation [7]. The ability of IL-8 to amplify neutrophil functions underscores its central role in the inflammatory process. Mastitis, defined as the inflammation of the mammary gland, is among the most debilitating diseases in all dairy species, including cattle [8,9,10]. In accordance with Regulation (EC) No 853/2004 [11], raw cow’s milk must not exceed a total bacterial count of 100,000 CFU/mL and a somatic cell count of 400,000 cells/mL. These thresholds are expressed as a “moving geometric mean”, calculated over a specified number of samples. The somatic cell count (SCC) serves as a global indicator of udder health in dairy animals and indirectly monitors milk quality [12,13]. Somatic cells in milk encompass mammary epithelial cells and various immune cell populations, including lymphocytes, polymorphonuclear cells (PMNs), and macrophages (MAC) [14,15,16]. Each cell type contributes differently to the inflammatory response, with lymphocytes regulating immune responses, PMNs defending against bacteria, and MACs involved in nonspecific immunity. In healthy cow milk, macrophages predominate, playing a pivotal role in the innate immune system by swiftly engulfing microbial invaders [17]. They release chemical messengers that attract PMNs to the infection site, where both cell types then phagocytose and eradicate microorganisms [18,19]. During ongoing infections, PMNs are recruited in large numbers, and comprise up to 92% of the cells in mastitis milk; they eliminate pathogens through oxidative and non-oxidative mechanisms [20,21]. Lymphocytes, which are essential to the response of the specific immune system, recognize antigens via specific membrane receptors [15]. Initially confused with macrophages, mammary epithelial cells, which are responsible for milk production, serve as the primary defense in mammary glands and potentially contribute to neonatal immunity in various species [20]. These cells are characterized by a specific mRNA and protein content, further confirming their identity [21]. Milk with an elevated somatic cell count (SCC) experiences compositional alterations primarily due to enhanced proteolytic activity, which detrimentally impacts its technological properties, including coagulation ability and cheese yield [22]. In the context of mastitis, particularly subclinical mastitis, there is an increase in serum protein concentrations at the expense of caseins, resulting in a decrease in the casein index. Among the various proteolytic enzymes present, plasmin activity is significantly elevated, leading to the breakdown of casein and subsequent impairment of the milk’s coagulation properties. Furthermore, lactose acts as an osmotic regulator, influencing the distribution of ions in milk, including sodium (Na), potassium (K), and chloride (Cl), which can further affect its functional characteristics [23]. Proteolytic activity, often attributed to PMNs, particularly impacts the casein fraction. It is believed that somatic cell counts in the milk of healthy cows are below 200,000 cells/mL [24]. An increase of more than 200,000 somatic cells per milliliter of milk, characteristic of the subclinical form of mastitis, can be typically detected by employing laboratory methods [25,26,27]. Heringstad et al. [28] demonstrated that selecting against mastitis leads to favorable responses in other diseases, such as ketosis and retained placenta, suggesting a general robustness or reduced susceptibility to disease. Additionally, De La Paz [29] found that cows with high antibody counts and cell-mediated immune responses have a lower risk of developing several diseases, including mastitis, ketosis, metritis, and retained placenta compared to cows identified as low responders. This highlights the importance of the immune response in disease resistance across the various health conditions in dairy cows.

Metabolic disorders involve disruptions to one or more metabolic processes; these encompass the release and conversion of metabolites utilized in production processes or eliminated as waste [30,31]. The dysfunction or physiological imbalance in these processes is linked to the development of metabolic diseases or disorders [32,33]. Ketosis is a condition that most commonly arises due to energy deficiencies, known as a negative energy balance (NEB), leading to abnormal metabolic processes in cows from calving to the end of the first two months of lactation. The main method of diagnosing ketosis, besides its clinical symptoms, is the determination of blood biochemical markers. During the course of the disease, there is an increase in the content of ketone bodies and free fatty acids, and a decrease in cholesterol and glucose levels [31]. Due to the systemic nature of metabolic disorders, they increase the risk of other conditions such as ruminal bloat, retained placenta, displacement of the abomasum, mastitis, and metritis [30,34]. The occurrence of postpartum paresis in dairy cows increases the risk of mastitis, retained placenta, metritis, abomasal displacement, calving difficulties, and ketosis. Ketosis, or fatty liver syndrome, often results from the occurrence of one or several conditions in cows, such as postpartum paresis, abomasal displacement, retained placenta, and calving difficulties [33]. Mastitis as an inflammatory condition of the mammary gland, induces systemic inflammation and immunological stress in affected cows [9]. This stress response can disrupt the normal metabolic processes, including glucose metabolism, contributing to the development of metabolic disorders such as ketosis or acidosis [31,35]. During the peripartum period, dairy cows commonly experience a negative energy balance [36,37]. This condition arises because the energy demands of late pregnancy and the onset of lactation exceed the energy intake, leading to an energy deficit. As a result, the immune system is compromised, increasing the cows’ susceptibility to infections. High-producing dairy cows, in particular, face greater energy demands. To meet these demands, they mobilize body fat reserves, releasing NEFAs into the bloodstream. This extensive mobilization can cause significant metabolic stress on the liver and impair the function of immune cells [38].

This period of negative energy balance is also associated with a dysfunctional immune system and an elevated inflammatory response. The stress of parturition, coupled with the metabolic and endocrine changes during the transition period, contributes to the modulation of pathways that regulate metabolism, immune status, and hormonal balance [39]. Immunosuppression is particularly evident in early lactation, a time when cows are most vulnerable to clinical mastitis. Mastitis is predominantly caused by opportunistic pathogens, indicating that the immune system fails to prevent the entry and proliferation of these pathogens within the mammary gland, leading to inflammation and infection in freshly calved cows. Mastitis caused by *Staphylococcus aureus* and *Streptococcus agalactiae* can be understood in terms of their pathogenicity and the immunological challenges faced by the host. Both bacteria are classified as infectious microorganisms with the mammary gland serving as their primary reservoir. Their ability to cause mastitis is linked to several factors. These pathogens have evolved specific virulence factors that enhance their colonization and persistence within mammary tissue, including the production of biofilms, toxins, and enzymes that facilitate tissue invasion and immune evasion [40]. In healthy cows, the immune system effectively controls the entry of pathogens into the mammary gland. However, factors such as stress, hormonal changes during the periparturient period, and compromised immune function can weaken this response, allowing these infectious microorganisms to invade, proliferate, and trigger inflammation [23]. Although these pathogens primarily reside in the mammary gland, they can also be transmitted between cows through direct contact or contaminated equipment, further exacerbating the mastitis incidence. Once established, these pathogens elicit a significant inflammatory response, characterized by increased somatic cell counts and the release of pro-inflammatory cytokines. This inflammation not only contributes to the clinical signs of mastitis but also leads to detrimental effects on milk production and quality [41].

This study aimed to provide insights into how IL-8 can be used as an indicator of both local (udder) and systemic (metabolic) inflammation and its interactions with the key metabolic enzymes related to liver function and energy balance in dairy cows. This study was designed to elucidate how IL-8 functions as a dual-purpose biomarker for monitoring both local inflammatory responses in the udder and the broader systemic metabolic conditions in dairy cows. By correlating Interleukin-8 with the somatic cell count, gamma-glutamyltransferase, aspartate aminotransferase, non-esterified fatty acids, and beta-hydroxybutyrate, the study aimed to delineate IL-8’s potential for reflecting not only udder health but also liver function and metabolic balance.

## 2. Results

The SCC is commonly used as an indicator of udder health in dairy cows, with changes in SCC levels reflecting the physiological changes or inflammatory responses in the mammary gland. The study categorized the SCC into five classes to investigate their association with various biomarkers in dairy cows. The SCC classes were defined as follows: Class I (<100,000 cells/mL), Class II (100,000–200,000 cells/mL), Class III (201,000–400,000 cells/mL), Class IV (401,000–1,000,000 cells/mL), and Class V (>1,000,000 cells/mL) [14,42].

The milk yield showed a significant decreasing trend across the SCC classes. Group I (SCC < 100,000 cells/mL) had the highest milk yield at 32.45 kg/d, which increased to 35.14 kg/d in Group II (SCC 100,000–200,000 cells/mL). However, from Group III onwards, a consistent decline was observed: 34.47 kg/d in Group III (SCC 201,000–400,000 cells/mL), 29.147 kg/d in Group IV (SCC 401,000–1,000,000 cells/mL), and the lowest at 25.083 kg/d in Group V (SCC > 1,000,000 cells/mL). The differences in the milk yield between the SCC classes were statistically significant (*p* ≤ 0.01), indicating an inverse relationship between the SCC levels and milk production (Table 1).

The protein percentage in milk showed a slight variation across the SCC classes. Group I exhibited the highest protein percentage at 3.67%. In Group II, the protein percentage decreased marginally to 3.54%, and in Group III, it further declined to 3.47%. A minor increase to 3.54% was observed in Group IV, and the percentage was the highest in Group V at 3.74%. These changes in protein percentage were statistically significant (*p* ≤ 0.01) (Table 1). The fat percentage in milk decreased significantly with higher SCC classes. Group I had the highest fat percentage at 3.95%, which decreased to 3.74% in Group II, 3.55% in Group III, 2.96% in Group IV, and 2.84% in Group V. The differences in fat percentage among the SCC classes were statistically significant (*p* ≤ 0.01), indicating an inverse relationship between the SCC levels and milk fat content (Table 1). The body condition score (BCS) showed a significant decline across the SCC classes. Group I had the highest BCS at 3.35, which decreased to 3.30 in Group II, 3.28 in Group III, 2.65 in Group IV, and 2.35 in Group V. The decrease in BCS was statistically significant (*p* ≤ 0.01), suggesting that higher SCC levels are associated with a deteriorating body condition. IL-8 concentrations increased significantly with higher SCC levels. Group I had the lowest IL-8 concentration at 0.6 ng/mL. This value increased to 1.1 ng/mL in Group II, 1.5 ng/mL in Group III, 8.2 ng/mL in Group IV, and 17.5 ng/mL in Group V. The differences in IL-8 concentrations among the SCC classes were highly significant (*p* ≤ 0.01), reflecting a strong positive correlation between the SCC levels and the inflammatory response. The data indicate that the milk yield decreases significantly with an increasing SCC, while the protein and fat percentages decline as the SCC increases, with the protein percentage showing a less pronounced trend. The BCS also decreased significantly with higher SCC levels, indicating deteriorating cow health. IL-8 concentrations rose substantially with an increasing SCC, indicating a strong inflammatory response. All the observed differences between the SCC classes for these parameters were statistically significant (*p* ≤ 0.01).

The analysis revealed significant differences in biomarker concentrations across the SCC classes (Table 2). The lysozyme (LZ) levels displayed a clear pattern across the SCC classes, progressively increasing from Class I to Class V. The differences were statistically significant (*p* < 0.001), indicating a strong association between the SCC levels and LZ concentrations. The percentage difference between Class I and Class V for LZ was 173.3%. The lactoferrin (LF) concentrations also exhibited variability across the SCC classes, albeit with a less pronounced trend. The statistical analysis (*p* < 0.05) suggests a borderline significant Spearman correlation coefficient between the LF levels and SCC. The percentage difference between Class I and Class V for LF was 34.8%. The beta-lactoglobulin (BLG) levels demonstrated distinct patterns across the SCC classes, with significant differences observed between the classes (*p* < 0.001). The BLG concentrations tended to increase with SCC classes I–V, indicating a potential association with udder health status. The percentage difference between Class I and Class V for BLG was 94.1%. The lactoperoxidase (LP) concentrations exhibited a significant variation across the SCC classes, with concentrations notably escalating in the higher SCC classes.

There was a statistically significant positive correlation between the SCC class and BLG concentration (r = 0.482, *p* < 0.01), indicating that as the SCC class increases, the concentration of BLG tends to increase as well. A statistically significant positive correlation was also observed between the SCC class and LP concentration (r = 0.412, *p* < 0.01), suggesting that higher SCC classes are associated with higher levels of LP. The LZ concentration showed a positive correlation with the SCC class (r = 0.135, *p* < 0.01). The LF concentration also displayed a weak positive correlation (r = 0.008, *p* > 0.05), although not statistically significant. There were weak positive correlations between LF and LP (r = 0.096, *p* < 0.05), LZ and LF (r = 0.291, *p* < 0.01), and LZ and LP (r = 0.113, *p* < 0.05). The BLG concentrations showed no significant correlation with the LZ, LF, or LP concentrations (Table 3).

The study revealed significant correlations between interleukin-8 and specific whey proteins, underscoring IL-8’s role in the inflammatory response within the mammary gland. IL-8 demonstrated a statistically significant positive correlation with LZ (r = 0.552, *p* < 0.01), BLG (r = 0.612, *p* < 0.01), and LP (r = 0.587, *p* < 0.01). These significant correlations suggest that elevated IL-8 levels are closely associated with increased concentrations of LZ, BLG, and LP, highlighting IL-8’s potential influence on these whey proteins during inflammatory processes.

Across the SCC classes, the glucose levels exhibited marginal variations. The percentage differences between consecutive SCC classes ranged from −10.94% to 9.17%, although these were statistically insignificant (*p* = 0.067). Conversely, the NEFA and BHBA concentrations showed substantial variations across the SCC classes. The NEFA concentrations notably increased with escalating SCC classes, with the percentage differences ranging from −18.49% to 283.75%. Similarly, the BHBA concentrations exhibited significant increases for the higher SCC classes, with the percentage differences ranging from −14.82% to 103.62%. These differences were statistically significant (*p* < 0.001), indicating a robust association between the SCC levels and NEFA/BHBA concentrations. The GGTP and AspAT levels also displayed marked variations across the SCC classes. Both biomarkers exhibited substantial increases for the higher SCC classes, with the percentage differences ranging from 0.59% to 511.67% for GGTP and from 3.92% to 60.96% for AspAT. These differences were statistically significant (*p* < 0.001), highlighting a strong association between the SCC levels and liver function biomarkers. Overall, while the glucose levels showed minimal changes across the SCC classes, the NEFA, BHBA, GGTP, and AspAT concentrations exhibited significant variations, suggesting their potential utility as indicators of the metabolic health and liver function in dairy cows (Table 4).

A statistically significant positive correlation was found between the SCC class and NEFA concentration (r = 0.503, *p* < 0.01), indicating that the higher SCC classes tend to be associated with elevated NEFA levels. Significant negative correlations were observed between the SCC class and both the glucose (r = 0.045, *p* > 0.05) and BHBA concentrations (r = 0.064, *p* > 0.05), indicating a potential inverse relationship between the SCC and these metabolic parameters. A strong positive correlation was observed between the SCC class and GGTP and AspAT concentration (r = 0.735, *p* < 0.01; r = 0.625, *p* < 0.01), suggesting that the higher SCC classes are associated with an increased GGTP and AspAT activity, indicating potential liver damage or dysfunction. The AspAT concentration showed no significant correlation with the SCC class or other metabolic parameters (Table 5). IL-8 showed a strong positive correlation with the SCC class (r = 0.712, *p* < 0.01). This indicates that higher levels of IL-8 are associated with higher SCC classes, reflecting a robust inflammatory response in the mammary gland. A moderate positive correlation was found between IL-8 and GGTP (r = 0.703, *p* < 0.01), as well as with AspAT (r = 0.742, *p* < 0.01).

The Spearman correlation matrix in Table 6 reveals several significant associations between the somatic cell count (SCC), metabolic markers, body condition, and milk yield, providing insights into the physiological and metabolic interactions within the dataset (Table 6). A strong negative correlation was observed between the SCC and BCS (r = −0.550, *p* < 0.01), suggesting that cows with an elevated SCC tend to have lower body condition scores. This inverse relationship may indicate the systemic metabolic burden associated with elevated somatic cell counts, where the immune activation and inflammation could be linked to a poor body condition. The correlation between the SCC and NEFAs is notably strong and positive (r = 0.650, *p* < 0.01), indicating that a higher SCC is associated with elevated non-esterified fatty acid levels, a marker of a negative energy balance. This relationship suggests that cows with an increased SCC may be experiencing greater metabolic stress, likely due to energy deficits that trigger fat mobilization. Liver enzymes show very strong positive correlations with the SCC, with GGTP (r = 0.792, *p* < 0.01) and AspAT (r = 0.782, *p* < 0.01) both being significantly associated with higher somatic cell counts. These results imply a connection between the SCC and liver function, possibly reflecting hepatic stress or damage in cows with elevated SCC levels, which could be a consequence of inflammatory processes or metabolic imbalances. The moderate negative correlation between the BCS and NEFAs (r = −0.55, *p* < 0.01) indicates that cows with lower body condition scores exhibit higher NEFA levels, a pattern commonly associated with a negative energy balance. Similarly, the BCS and AspAT showed a moderate negative correlation (r = −0.49, *p* < 0.01), suggesting that cows in a poorer body condition experience a higher AspAT activity, pointing to potential liver dysfunction or metabolic strain in cows with energy deficits. A moderate positive correlation between NEFAs and AspAT (r = 0.46, *p* < 0.01) further supports the connection between an energy imbalance and liver function, suggesting that cows with elevated NEFAs, a marker of fat mobilization, tend to have higher liver enzyme levels, likely reflecting metabolic stress. Additionally, the strong positive correlation between NEFAs and IL-8 (r = 0.67, *p* < 0.01) suggests a link between metabolic dysregulation and systemic inflammation, with higher NEFA levels associated with increased IL-8, an inflammatory cytokine.

In summary, these correlations underscore the significant relationships between the metabolic health, liver function, body condition, and somatic cell count. An elevated SCC is strongly associated with metabolic markers such as NEFAs and liver enzymes, indicating a possible metabolic and hepatic strain in cows experiencing immune activation. These findings suggest that metabolic stress and liver dysfunction may play a crucial role in influencing the overall cow health and productivity.

## 3. Discussion

Interleukin-8 is a key pro-inflammatory cytokine involved in the immune response to mastitis in dairy cows. The data from this study show a significant increase in IL-8 concentrations as the SCC class increases, from 0.6 ng/mL in Class I to 17.5 ng/mL in Class V. This trend reflects IL-8’s role in mediating the inflammatory response in the mammary gland. IL-8 functions primarily as a chemotactic factor for neutrophils. It attracts neutrophils to the site of infection and activates them to release inflammatory mediators [43].

The observed patterns for the whey protein concentrations across the SCC classes reflect the dynamic nature of the mammary gland’s immune response to infection and inflammation. For instance, the progressive increase in LZ levels with the higher SCC classes suggests a heightened immune response to microbial invasion, attributed to lysozymes’ antimicrobial properties [44]. Similarly, the variations in LF and BLG concentrations across the SCC classes highlight the complex interplay between the immune function and milk synthesis in the mammary gland. Lactoferrin, an iron-binding protein with antimicrobial properties, is produced by the epithelial cells in response to infection and inflammation, explaining its elevated levels in milk with higher SCCs [45,46]. On the other hand, beta-lactoglobulin, a major whey protein, may reflect the changes in the mammary epithelial cell function and milk synthesis capacity in response to inflammatory stimuli [47,48]. BLG, a member of the lipocalin family, which includes, among others, fatty acid-binding proteins and bacterial metalloprotease inhibitors [49], could have a role in the innate immune response of the mammary gland. The significant variation in the LP concentrations across the SCC classes further emphasizes the SCC’s role as a predictor of mammary gland health and immune status [50]. Lactoperoxidase, an enzyme involved in the innate immune defense of the mammary gland, is secreted by the epithelial cells in response to a microbial challenge, indicating its potential utility as a biomarker for mastitis detection [19]. Conversely, the lower SCC classes exhibited diminished whey protein concentrations, suggesting a healthier udder environment and reduced inflammatory response [45]. Additionally, the substantial differences in the whey protein concentrations between SCC Class I and V underscore the influence of udder health on milk protein composition [50,51]. These findings highlight the intricate interplay between the immune function, metabolic processes, and milk synthesis in dairy cows, necessitating comprehensive management strategies for optimal udder health and milk quality [19].

Metabolic disorders such as ketosis profoundly impact the milk composition and udder health in dairy cows [33,52,53,54]. Additionally, Wang et al. [55] reported that microbial populations may be related to ketosis by affecting the short-chain fatty acid metabolism and BHB accumulation even in cows with adequate feed intake in the early postpartum.

Furthermore, the intricate relationship between fatty acids and milk proteins, particularly BLG, underscores the complexity of the milk composition regulation [56,57]. BLG’s ability to bind to fatty acids influences fatty acid metabolism and, consequently, the milk protein synthesis. BLG concentrations tended to increase with the higher SCC classes, indicating a potential association with the udder health status. The percentage difference between Class I and Class V for BLG was 94.1%. The interactions between β-lactoglobulin, the somatic cell count, and the enzymes, aspartate aminotransferase and gamma-glutamyl transferase, unveil the complex dynamics within the dairy cow physiology. First, elevated SCC levels often signal inflammation and immune activation in the mammary gland. Studies have shown that in SCC class V, indicative of heightened inflammation, the BLG levels significantly increase. This suggests a possible association between an elevated SCC and alterations in the milk protein synthesis, with BLG serving as a biomarker for the udder health status. Furthermore, the presence of AspAT and GGTP, enzymes that are often indicative of liver damage or dysfunction, alongside the high SCC levels in SCC class V, adds another layer of complexity. The simultaneous increase in BLG levels emphasizes the potential link between metabolic disorders such as ketosis and the disruptions in liver function, which impact the milk protein composition [37].

The marginal variations observed in the glucose levels across the different SCC classes suggest that the SCC may not directly influence the glucose metabolism. Glucose serves as a vital energy source in cows and is primarily derived from dietary carbohydrates. The minimal changes in the glucose levels indicate that the SCC may not directly impact the glucose utilization or production in the body.

Conversely, the substantial variations in the NEFAs and BHBA concentrations across the SCC classes express their crucial role as metabolic indicators. NEFAs and BHBA are vital components of the metabolic profile used in diagnosing the metabolic diseases in dairy cows [33,35]. For NEFAs, the reference values range from 0.25 to 0.6 mmol/L, with levels exceeding 0.6 mmol/L indicating metabolic stress [52,53,54]. BHBA concentrations, on the other hand, are considered normal at levels below 0.6 mmol/L. Subclinical ketosis is often diagnosed when the BHBA levels range between 0.51 to 1.2 mmol/L, while concentrations surpassing 2.5 mmol/L indicate clinical ketosis [33,58]. The significant increases observed in NEFAs and BHBA concentrations for the higher SCC classes suggest a robust association between the SCC levels and metabolic disturbances, highlighting the SCC’s utility as an indicator of metabolic health.

Furthermore, the marked variations in the GGTP and AspAT levels across the SCC classes indicate potential liver dysfunction. Aspartate aminotransferase is an enzyme primarily localized in the mitochondria of cells. This is significant in diagnostic contexts because when minor cell damage occurs, the bloodstream primarily contains cytoplasmic enzymes; however, as cell damage progresses, mitochondrial enzymes such as AspAT are also released into the bloodstream [59,60]. Elevated AspAT concentrations in the bloodstream can occur due to various factors such as bruising, trauma, necrosis, infection, or liver or muscle neoplasia [30]. Additionally, hepatic disorders may manifest across the spectrum of severity, with the AspAT levels rising in response to conditions like liver damage or dysfunction [61]. In this study, there was a significant positive correlation between IL-8 and AspAT levels (r = 0.625, *p* < 0.01), suggesting that increased IL-8 concentrations are associated with higher AspAT levels. This relationship implies that as mastitis severity increases, the associated systemic inflammation may extend to liver tissue, contributing to an increased AspAT activity. It is noteworthy that the hepatic congestion resulting from right ventricular dysfunction, often associated with heart failure, can also contribute to the increased AspAT levels. In cattle, the reference values for AspAT typically range from 58 to 100 U/L [62]. GGTP is an enzyme involved in various physiological processes, particularly in the metabolism of glutathione, a vital antioxidant in the body. GGTP is primarily found on the outer surface of cell membranes and is also present in the bloodstream. In dairy cows, GGTP serves as a marker for liver and biliary tract function [62,63]. The elevations in GGTP levels in the bloodstream often indicate liver damage or dysfunction, as the enzyme is released into the circulation when the liver cells are damaged or stressed. In this study, the IL-8 levels were also positively correlated with the GGTP concentrations (r = 0.735, *p* < 0.01). This correlation suggests that IL-8, a marker of local inflammation in the mammary gland, may be linked to broader systemic effects that include liver dysfunction. The rise in GGTP with increasing IL-8 levels indicates that mastitis-induced inflammation might contribute to or exacerbate liver damage, as GGTP is released into the bloodstream when liver cells are damaged or stressed. The reference values for GGTP in cattle typically range from 22 to 64 U/L [62]. Additionally, GGTP plays a role in the catabolism of glutathione as well as the catalysis of low-density lipoprotein oxidation [63]. The observed correlations between IL-8 and both AspAT and GGTP underscore the interconnected nature of the mastitis, inflammation, and systemic health in dairy cows. In groups 4 and 5, a low fat-to-protein ratio (FPR) was observed, with values falling below 1. Prior research indicates that a low FPR is often indicative of subclinical ruminal acidosis (SARA), a metabolic condition that can adversely affect the lipogenesis within the mammary gland. Although our investigation did not directly diagnose SARA, the documented low FPR may suggest the presence of this disorder. Furthermore, the study by Puppel et al. [31] demonstrates that the AspAT activity is elevated by nearly 1.90-fold in cows diagnosed with acidosis compared to those exhibiting ketosis. The incorporation of enzymatic markers such as AspAT and GGTP may facilitate the reduction in the sampling frequency required for laboratory analyses, thereby expediting the diagnosis of metabolic disorders. The AspAT activity in serum has been recognized as a robust indicator of acidosis, while GGTP may be implicated in the pathophysiology of ketosis.

High IL-8 levels correlate with increased AspAT and GGTP levels, indicating that severe mastitis can be accompanied by significant systemic inflammation and potential liver damage. Monitoring the IL-8 levels could provide insights into mastitis severity and the associated systemic effects on liver function. Both mastitis and ketosis are associated with a negative energy balance in dairy cows [64]. Mastitis-induced inflammation can decrease the feed intake and increase the energy requirements for the immune function, exacerbating the NEB commonly observed in early lactation, which is a predisposing factor for ketosis [65]. Mastitis-related inflammation and stress can impact liver function, affecting its ability to metabolize fatty acids and glucose [66]. The stress response associated with mastitis, characterized by elevated cortisol levels, can impair the insulin sensitivity and exacerbate the development of ketosis [67]. Chronic stress can further disrupt metabolic homeostasis and increase the risk of ketosis. Additionally, Chirivi et al. [68] suggest that bacteremia or endotoxemia, systemic inflammation, and pain may play crucial roles in clinical ketosis pathogenesis.

## 4. Materials and Methods

### 4.1. Data Collection

The study was conducted at the Warsaw University of Life Sciences’ (WULS) experimental dairy farm, which maintains a herd of approximately 380 cows within a free-stall housing system. As part of the routine health monitoring procedures, all cows were examined, with each cow (muliparous) being categorized into one of five distinct groups based on their somatic cell count (SCC) levels. The SCC classification criteria were as follows: Group I (<100,000 cells/mL; n = 45), Group II (100,000–200,000 cells/mL; n = 62), Group III (201,000–400,000 cells/mL; n = 52), Group IV (401,000–1,000,000 cells/mL; n = 73 and Group V (>1,000,000 cells/mL; n = 56). This systematic categorization allowed for a comprehensive investigation into the associations between the SCC and various metabolic profile parameters. Strict criteria were implemented to select the cows for the study, ensuring that only the animals free from hoof issues, such as sole ulcers, or other health conditions that could influence the somatic cell count levels were included. Regular veterinary examinations were conducted to assess the overall health of the selected animals. These examinations confirmed that all the cows included in the study were primarily free from health issues, with the exception of mastitis. This careful selection process allowed us to conclude that the observed changes in the SCC were predominantly linked to the inflammatory processes occurring in the mammary gland.

The SCC values were categorized into five groups (Group I: <100,000 cells/mL; Group II: 100,000–200,000 cells/mL; Group III: 201,000–400,000 cells/mL; Group IV: 401,000–1,000,000 cells/mL; Group V: >1,000,000 cells/mL) to simplify the analysis and enhance the interpretability of the results. This approach facilitated a clearer comparison of the metabolic profiles across the different levels of udder inflammation and helped in understanding the relationship between the SCC levels and various metabolic parameters. While categorizing a continuous variable into discrete groups does lead to some loss of precision, it provides practical benefits in terms of data analysis and interpretation. To address the potential concerns about the reduced statistical power and accuracy, we ensured a sufficiently large sample size within each category to maintain robust comparisons. This approach allowed us to identify the significant trends and associations effectively.

The body condition score (BCS) was assessed by using the BCS-5 method described by Edmonson et al. [69], which evaluates the fat reserves based on the visual and manual inspection of specific anatomical sites, including the ribs, spine, and pelvis. The scale ranges from 1 to 5, with 1 indicating severe emaciation and negligible fat reserves, and 5 representing excessive adiposity with prominent fat deposits throughout the body.

The diets administered to the cows were balanced in accordance with the INRA system’s recommendations. The feeding regime centered around a total mixed ration (TMR) diet, provided ad libitum to the cows. The composition of the TMR (kg/d DM) included maize silage (11.05), alfalfa silage (3.50), corn silage (2.50), soybean meal (2.50), pasture ground chalk (0.20), salt (0.05), rapeseed meal (2.10), and magnesium oxide (0.06). The additional parameters characterizing the TMR were as follows: total dry matter (DM) content (21.20 kg), daily intake (19.90 kg), net energy for lactation (Mcal/kg) (1.75), average milk production (37.02 kg), unit of milk production balance (%) (3.45), protein digested in the small intestine when rumen-fermentable nitrogen is limiting (2.51), and protein digested in the small intestine when rumen-fermentable energy is limiting (2.23).

Samples of milk and blood were collected from the cows at 60 ± 5 days postpartum, resulting in a total of 288 samples of both milk and blood. The study chose 60 days in milk (DIM) as the time point for the sample collection, corresponding to the peak of lactation for the herd. This timing was selected to capture the potential fluctuations in the somatic cell count levels and metabolic profile parameters during this critical phase of the lactation cycle when the metabolic demands are high. To ensure representative sampling across the SCC categories, explicit criteria were developed for selecting the cows at 60 DIM. This involved stratified sampling based on the SCC levels at this specific time point, with efforts made to achieve a balanced representation in each SCC category, including the extreme categories (<100,000 and >1,000,000 cells/mL).

The daily milk yield was recorded, and the individual milk samples were extracted for the subsequent compositional analyses. The milk samples, each comprising 250 mL, were collected from each cow during both the morning and evening milking sessions using specialized milk samplers. These samples were carefully transferred to sterile bottles and promptly transported to the Milk Testing Laboratory of Warsaw University of Life Sciences (WULS) for comprehensive compositional analysis. For the blood sample collection (at the same hour of the day from each animal, 60 ± 5 days postpartum), approximately 10 mL of blood was drawn from the cows via a jugular vein puncture using Vacuette tubes (Essen, Germany), containing potassium-EDTA (K3EDTA, 1.8 g/L of blood) as an anticoagulant. The blood samples were then subjected to centrifugation at 1800× *g* at 4 °C for 15 min. Following the centrifugation, the supernatant was promptly transported to the WULS Veterinary Centre for further analysis.

The cows delivered healthy, full-term calves without assistance, demonstrating effective management of their metabolic and energy challenges. The routine health monitoring procedures were integrated into the study protocol to collect data on the cows’ clinical mastitis status, other health indicators, and body condition scores. This comprehensive approach accounted for the potential confounding variables in the analyses, providing the context for interpreting the results.

### 4.2. Chemical Analyses

The fat, protein, lactose, and casein as well as density were determined using a Milko-Scan FT-120 analyzer (Foss Electric, Hillerød, Denmark). The cytological quality (somatic cell count: SCC) was established using a Somacount 150 analyzer (Bentley, Warsaw, Poland). The reference milk (reference material) for fat, protein, and the SCC, obtained from the National Animal Breeding Centre, was used to create the calibration curves for the analyzers.

The whey protein concentrations were determined using an Agilent 1100 Series RP-HPLC (Agilent Technologies, Waldbronn, Germany). Separations were performed at ambient temperature using a solvent gradient with a C18 300A Jupiter column (Phenomenex, Torrance, CA, USA). The chromatographic conditions were as follows: solvent A consisted of acetonitrile (Merck, Darmstadt, Germany), water (Sigma-Aldrich, Burlington, MA, USA), and trifluoroacetic acid (Sigma-Aldrich, Burlington, MA, USA), at a ratio of 70:930:1 (*v*/*v*/*v*), respectively. Solvent B consisted of acetonitrile, water, and trifluoroacetic acid at a ratio of 930:70:1 (*v*/*v*/*v*), respectively. The flow rate was 1.4 mL/min, and the detection wavelength was 220 nm. All the samples were analyzed in duplicate. The identification of the peaks as lactoferrin and lysozyme was confirmed by comparing them with the standards (Sigma-Aldrich, Burlington, MA, USA).

The levels of BHBA, NEFAs, AspAT, glucose, and GGTP (in blood plasma) were determined using a BS800M biochemical analyzer (PZ Cormay, Warsaw, Poland) at the WULS Veterinary Centre.

The cattle IL-8 (Interleukin-8) levels in blood plasma were determined using a NanoQuant Infinite M200Pro analyzer (Tecan Austria GmbH, Grödig, Austria) and a dedicated ELISA Kit (Cat#: MBS8801876, MyBioSource, Inc., San Diego, CA, USA). The assay principle of this kit was based on a Sandwich enzyme immunoassay. The microtiter plate provided in the kit was pre-coated with an antibody specific to IL-8. The standards or samples were added to the wells, followed by a biotin-conjugated antibody specific to IL-8. Subsequently, Avidin conjugated to Horseradish Peroxidase (HRP) was added and incubated. Upon the addition of the TMB substrate solution, a color change occurred in the wells containing IL-8, the biotin-conjugated antibody, and the enzyme-conjugated Avidin. The enzyme–substrate reaction was terminated with sulfuric acid solution, and the color change was measured spectrophotometrically at a wavelength of 450 nm ± 10 nm. The IL-8 concentration in the samples was determined by comparing the optical density (OD) of the samples to the standard curve. The ELISA kit used for the IL-8 analysis was specifically validated for bovine species (cattle). The kit’s sensitivity is 6.2 pg/mL. The intra-assay precision has a coefficient of variation (CV) of less than 8%, while the inter-assay precision has a CV of less than 10%.

### 4.3. Statistical Analyses

The data were statistically analyzed using the variance analysis (ANOVA) with the SCC group as the main analyzed factor. The Shapiro–Wilk test was used to verify the distribution of the milk and blood components. The *p* value obtained from the Shapiro–Wilk test was equal to 0.672. The tests were performed using IBM SPSS 23 software [70]. Only those interactions between the factors whose impact was statistically significant (*p* ≤ 0.01 or *p* ≤ 0.05), as determined after preliminary statistical analyses, were included in the study.

A Principal Component Analysis (PCA) was conducted to reduce the dimensionality of the dataset and identify the principal components that captured the most variance (Figure S1). The results indicate that the first principal component (PC1), associated with the SCC variable, explained 64.8% of the total variance, suggesting that it captured the most significant portion of the dataset’s variability. The second principal component (PC2), related to the yield, accounted for 11.4% of the variance, while the third principal component (PC3), associated with the BCS, explained 8.5% of the variance. The subsequent components, including PC4 (glucose levels), PC5 (NEFAs), and others, explained progressively smaller portions of the variance. Overall, the first few components (PC1 to PC3) were identified as the most critical, as they collectively accounted for the majority of the dataset’s variability, making them the key focus for analysis.

The methodology for conducting the correlation analysis using the Pearson and Spearman coefficients involves several key steps. First, the data collection phase consists of gathering a dataset that includes various metabolic and body condition variables. These variables are both continuous and ordinal in nature, which determines the appropriate correlation method. The variable selection is guided by the type of data, where the continuous variables that are assumed to have a normal distribution and exhibit linear relationships are analyzed using Pearson correlation, while the ordinal variables or those that do not necessarily follow a normal distribution and may have non-linear, monotonic relationships are analyzed using Spearman correlation. The Pearson correlation analysis was applied to variables such as glucose, IL-8, NEFAs, BHBA, GGTP, AspAT, protein, fat, LZ, LF, BLG, and LP. These variables were expected to follow a linear relationship and normal distribution. On the other hand, Spearman correlation was used for variables such as the yield and BCS, which may have ordinal or non-linear relationships. In the calculation process, the dataset was split into two groups: one for the Pearson correlation and one for the Spearman correlation. The results were then stored in two separate correlation matrices: a Pearson correlation matrix for continuous variables with assumed linear relationships, and a Spearman correlation matrix for ordinal or non-linear relationships. The strength of the relationships between the variables was interpreted based on the correlation coefficient values. The strength of the relationships between the variables was interpreted based on the correlation coefficient values. If |r| is less than 0.2, it indicates no linear relationship. Values between 0.2 and 0.4 suggest a weak correlation, while values between 0.4 and 0.7 indicate a moderate correlation. A coefficient between 0.7 and 0.9 reflects a fairly strong correlation, and if |r| is greater than 0.9, it is considered a very strong correlation.

## 5. Conclusions

This study aimed to provide insights into how IL-8 can be used as an indicator of both local (udder) and systemic (metabolic) inflammation and its interactions with the key metabolic enzymes related to the liver function and energy balance in dairy cows.

The analysis confirmed that the IL-8 levels significantly increased with the higher SCC classes, demonstrating IL-8’s effectiveness as an indicator of udder inflammation. A strong positive correlation between IL-8 and the SCC supported IL-8’s role in reflecting the inflammatory processes in the mammary gland. IL-8 was positively correlated with NEFAs and BHBA, indicating that higher IL-8 levels are associated with the increased concentrations of these metabolites, which are markers of ketosis and a negative energy balance. Further, IL-8 showed positive correlations with GTTP and AspAT, indicating that higher IL-8 levels are linked to the increased activities of these liver enzymes.

The variations in the metabolic parameters, including NEFAs, BHBA, GGTP, and AspAT, across the SCC classes underscored the association between the elevated SCC levels and metabolic dysregulation in dairy cows. The data revealed significant increases in the NEFA and BHBA concentrations with the higher SCC classes, reflecting a heightened metabolic stress response and indicating a potential state of negative energy balance and ketosis. Concurrently, the GGTP and AspAT levels exhibited a significant elevation in the higher SCC classes, suggesting a correlation between an increased SCC and liver dysfunction, as these enzymes are biomarkers for hepatic injury and impaired liver function. These findings highlight the interrelated nature of the inflammatory responses and metabolic disturbances in dairy cattle, emphasizing that an elevated SCC not only signifies udder inflammation but also correlates with systemic metabolic alterations indicative of ketosis and liver damage.

## Figures and Tables

**Table 1:** The cows’ characteristics (milk yield, protein, fat, BCS, and Il-8).

SCC Class											
	Group I (n = 45)		Group II (n = 62)		Group III (n = 52)		Group IV (n = 73)		Group V (n = 56)		*p*-Value
	LSM	SEM	LSM	SEM	LSM	SEM	LSM	SEM	LSM	SEM	
Milk parameters											
Milk Yield [kg/d]	32.45 ^A,B,C,D^	1.412	35.14 ^A,E,F^	1.527	34.47 ^B,G,H^	1.308	29.147 ^C,E,G,I^	1.333	25.08 ^D,F,H,I^	1.212	*p* ≤ 0.01
Protein [%]	3.67 ^a,B,C,d^	0.154	3.54 ^a,e,F^	0.139	3.47 ^B,e,G,H^	0.101	3.54 ^C,G,I^	0.162	3.74 ^d,F,H,I^	0.141	*p* ≤ 0.01
Fat [%]	3.95 ^A,B,C,D^	0.114	3.74 ^A,E,F^	0.116	3.55 ^B,G,H^	0.120	2.96 ^C,E,G,I^	0.143	2.84 ^D,F,H,I^	0.159	*p* ≤ 0.01
BCS	3.35 ^a,B,C^	0.051	3.30 ^D,E^	0.042	3.28 ^a,F,G^	0.044	2.65 ^B,D,F,H^	0.032	2.35 ^C,E,G,H^	0.025	*p* ≤ 0.01
IL-8 [ng/mL]	0.6 ^A,B,C,D^	0.021	1.1 ^A,E,F,G^	0.082	1.5 ^B,E,H,I^	0.113	8.2 ^C,F,H,J^	0.277	17.5 ^D,G,I,J^	0.457	*p* ≤ 0.01

SCC class. I: <100,000 cells/mL; II: 100,000–200,000 cells/mL; III: 201,000–400,000 cells/mL; IV: 401,000–1,000,000 cells/mL; V: >1,000,000 cells/mL; SCC, somatic cell count; BCS, body condition score; IL-8, Interleukin-8; LSM, least square mean; SEM, standard error of LSM; values with the same letters in the row differ significantly: upper case at *p* ≤ 0.01; small case at *p* ≤ 0.05.

**Table 2:** Changes in the concentration of the whey profile for varying levels of the SCC.

		SCC Class					*p*-Value
		Group I	Group II	Group III	Group IV	Group V	
LZ [µg/L]	LSM	18.82 ^A,B,C^	17.64 ^D,E,F^	21.83 ^A,D,G,H^	35.92 ^B,E,G,I^	51.49 ^C,F,H,I^	0.000
	SEM	0.225	0.114	0.136	0.328	0.228	
LF [mg/L]	LSM	0.23 ^a^	0.24 ^b^	0.25 ^c^	0.24 ^d^	0.31 ^a,b,c,d^	0.05
	SEM	0.021	0.026	0.019	0.017	0.018	
BLG [g/L]	LSM	3.06 ^A,B,C,D^	3.7 ^A,E,f,G^	3.27 ^B,E,H,I^	3.88 ^C,f,H,J^	5.94 ^D,G,I,J^	0.000
	SEM	0.114	0.115	0.117	0.0136	0.117	
LP [mg/L]	LSM	0.26 ^a,B^	0.30 ^a,c,d,E^	0.26 ^c,F^	0.27 ^d,G^	1.28 ^B,E,F,G^	0.000
	SEM	0.011	0.012	0.011	0.013	0.017	

SCC class. I: <100,000 cells/mL; II: 100,000–200,000 cells/mL; III: 201,000–400,000 cells/mL; IV: 401,000–1,000,000 cells/mL; V: >1,000,000 cells/mL; SCC, somatic cell count; LSM, least square mean; SEM, standard error of LSM; LZ, lysozyme; LP, lactoperoxidase; LF, lactoferrin; BLG, beta-lactoglobulin; values with the same letters in the row differ significantly: upper case at *p* ≤ 0.01; small case at *p* ≤ 0.05.

**Table 3:** Pearson correlations between IL-8 and selected whey proteins.

	LZ	LF	BLG	LP	IL-8
LZ	1	0.291 **	0.007	0.113 **	0.552 **
LF		1	−0.072	0.096 *	0.129
BLG			1	0.064	0.612 **
LP				1	0.587 **
IL-8					1

SCC, somatic cell count; LZ, lysozyme; LP, lactoperoxidase; LF, lactoferrin; BLG, beta-lactoglobulin; IL-8, interleukin-8; ** correlation significant at a level of 0.01 (two-sided); * correlation significant at a level of 0.05 (two-sided).

**Table 4:** Changes in the metabolic profile for varying levels of the SCC.

		SCC Class					*p*-Value
		Group I	Group II	Group III	Group IV	Group V	
Glucose [mg/dL]	LSM	64.850	65.963	71.996	64.207	64.692	0.067
	SEM	2.147	2.114	2.116	2.221	2.203	
NEFAs [mmol/L]	LSM	0.383 ^A,B^	0.312 ^C,D^	0.400 ^E,F^	1.535 ^A,C,E,G^	1.626 ^B,D,F,G^	0.000
	SEM	0.022	0.022	0.021	0.019	0.018	
BHBA [mmol/L]	LSM	0.826 ^A,B^	0.719 ^C,D^	0.613 ^E,F^	1.251 ^A,C,E^	1.282 ^B,D,F^	0.000
	SEM	0.036	0.038	0.033	0.041	0.044	
GGTP [U/L]	LSM	26.067 ^A,B,C^	24.102 ^D,E,F^	147.193 ^A,D,G,H^	162.221 ^B,E,G^	163.114 ^C,F,H^	0.000
	SEM	1.222	1.306	1.284	1.265	1.241	
AspAT [U/L]	LSM	88.575 ^A,B,C,D^	78.098 ^A,E,F,G^	81.224 ^B,E,H,I^	130.842 ^C,F,H,J^	171.321 ^D,G,I,J^	0.000
	SEM	2.114	2.225	2.198	2.412	2.228	

SCC class. I: <100,000 cells/mL; II: 100,000–200,000 cells/mL; III: 201,000–400,000 cells/mL; IV: 401,000–1,000,000 cells/mL; V: >1,000,000 cells/mL; SCC, somatic cell count; LSM, least square mean; SEM, standard error of LSM; NEFAs, non-esterified fatty acids; BHBA, beta-hydroxybutyrate; GGTP, gamma-glutamyltransferase; AspAT, aspartate aminotransferase; values with the same letters in the row differ significantly: upper case at *p* ≤ 0.01; small case at *p* ≤ 0.05.

**Table 5:** Pearson correlations between IL-8 and the parameters of metabolic profiles.

	Glucose	NEFAs	BHBA	GGTP	AspAT	IL-8
Glucose	1	−0.193 **	−0.490 **	0.045	−0.061	0.241
NEFAs		1	0.702 **	0.408 **	−0.007	0.412 **
BHBA			1	−0.013	0.073	0.641 **
GGTP				1	0.714 **	0.703 **
AspAT					1	0.742 **
IL-8						1

NEFAs, non-esterified fatty acids; BHBA, beta-hydroxybutyrate; GGTP, gamma-glutamyltransferase; AspAT, aspartate aminotransferase; IL-8, interleukin-8; ** correlation significant at a level of 0.01 (two-sided).

**Table 6:** Sperman correlations between the SCC, IL-8, parameters of metabolic profiles, and whey proteins.

	SCC	SCC Class	Milk Yield	BCS	Glucose	NEFAs	BHBA	GGTP	AspAT	IL-8	LZ	LF	BLG	LP
SCC	1	0.720	−0.165	−0.550 **	0.004	0.650 **	0.218 *	0.792 **	0.782 **	0.764 **	0.366 *	0.056	0.347 **	0.632 **
SCC class		1	−0.090	−0.675 **	0.103	0.565 **	0.487 **	0.870 **	0.444 **	0.825 **	0.382 *	0.056	0.397 *	0.520 **
Milk yield			1	0.137 *	−0.207	−0.133 *	−0.028	−0.090	−0.165 *	−0.186 *	−0.120 *	−0.009	−0.169 *	−0.134 *
BCS				1	−0.03	−0.55 **	−0.27 **	−0.58 **	−0.49 **	−0.76 **	−0.37 *	−0.06	−0.31 *	−0.52 **
Glucose					1	−0.16	−0.41	0.13	−0.05	0.31 *	−0.03	0.03	0.11	0.11
NEFA						1	0.42 **	0.52 **	0.46 **	0.67 **	0.42 **	0.02	0.22 *	0.38 **
BHBA							1	0.18	0.33 *	0.54 **	0.20 *	0.02	0.05	0.19
GGTP								1	0.417	0.678 **	0.355 *	0.052	0.243 *	0.350 *
AspAT									1	0.64 **	0.32 *	0.01	0.27 *	0.46 **
IL-8										1	0.46 **	0.07	0.63 **	0.72 **
LZ											1	0.38 *	0.20 *	0.28 *
LF												1	−0.01	0.08
BLG													1	0.33 *
LP														1

SCC, somatic cell count; NEFAs, non-esterified fatty acids; BHBA, beta-hydroxybutyrate; GGTP, gamma-glutamyltransferase; AspAT, aspartate aminotransferase; IL-8, interleukin-8; LZ, lysozyme; LP, lactoperoxidase; LF, lactoferrin; BLG, beta-lactoglobulin; ** correlation significant at a level of 0.01 (two-sided); * correlation significant at a level of 0.05 (two-sided).

## Data Availability

All data generated or analyzed during the study are included within the article.

## Supplementary Materials

The following supporting information can be downloaded at https://www.mdpi.com/article/10.3390/ijms252011129/s1.
