# Supplementary material for: Assessing the Usefulness of Interleukin-8 as a Biomarker of Inflammation and Metabolic Dysregulation in Dairy Cows
Source: Int J Mol Sci. 2024 Oct 16;25(20):11129. doi: 10.3390/ijms252011129 (PMC11508773; doi:10.3390/ijms252011129)
Supplement: Supplementary file 1 [file ijms-25-11129-s001.zip › ijms-3223885-supplementary.pdf]

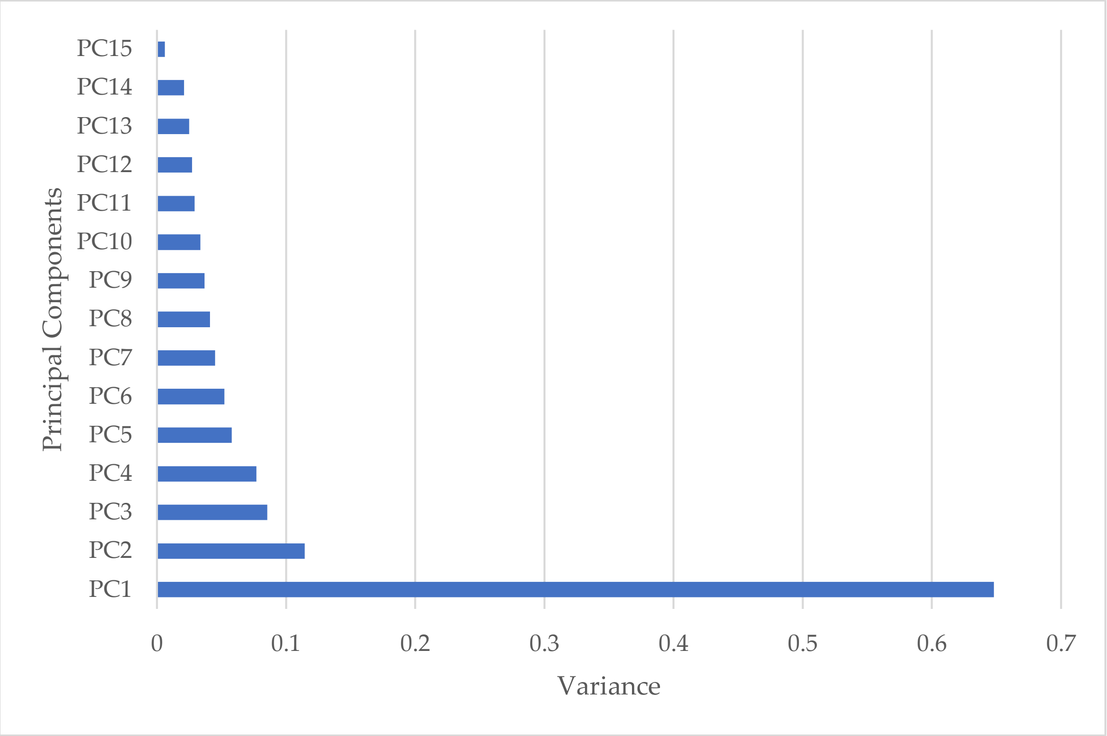

Supplementary Figure S1. Principal Components and Explained Variance. PC1: SCC, PC2: Yield, PC3: BCS, PC4: Glucose, PC5: NEFA, PC6: BHBA, PC7: GGTP, PC8: AspAT, PC9: IL-8, PC10: Protein, PC11: Fat, PC12: LZ, PC13: LF, PC14: BLG, PC15: LP.
